# Different Effects of sgRNA Length on CRISPR-mediated Gene Knockout Efficiency

**DOI:** 10.1038/srep28566

**Published:** 2016-06-24

**Authors:** Jian-Ping Zhang, Xiao-Lan Li, Amanda Neises, Wanqiu Chen, Lin-Ping Hu, Guang-Zhen Ji, Jun-Yao Yu, Jing Xu, Wei-Ping Yuan, Tao Cheng, Xiao-Bing Zhang

**Affiliations:** 1 State Key Laboratory of Experimental Hematology, Chinese Academy of Medical Sciences and Peking Union Medical College, Tianjin, China; 2 Institute of Hematology and Blood Disease Hospital, Chinese Academy of Medical Sciences and Peking Union Medical College, Tianjin, China; 3 Department of Medicine, Loma Linda University, Loma Linda, CA 92350, USA; 4 Center for Stem Cell Medicine, Chinese Academy of Medical Sciences, Tianjin, China; 5 Department of Stem Cell & Regenerative Medicine, Peking Union Medical College, Tianjin, China; 6 Collaborative Innovation Center for Cancer Medicine, Tianjin, China; 7 Tianjin Key Laboratory of Blood Cell Therapy and Technology, Tianjin, China

## Abstract

CRISPR-Cas9 is a powerful genome editing technology, yet with off-target effects. Truncated sgRNAs (17nt) have been found to decrease off-target cleavage without affecting on-target disruption in 293T cells. However, the potency of 17nt sgRNAs relative to the full-length 20nt sgRNAs in stem cells, such as human mesenchymal stem cells (MSCs) and induced pluripotent stem cells (iPSCs), has not been assessed. Using a GFP reporter system, we found that both 17nt and 20nt sgRNAs expressed by lentiviral vectors induce ~95% knockout (KO) in 293T cells, whereas the KO efficiencies are significantly lower in iPSCs (60–70%) and MSCs (65–75%). Furthermore, we observed a decrease of 10–20 percentage points in KO efficiency with 17nt sgRNAs compared to full-length sgRNAs in both iPSCs and MSCs. Off-target cleavage was observed in 17nt sgRNAs with 1-2nt but not 3-4nt mismatches; whereas 20nt sgRNAs with up to 5nt mismatches can still induce off-target mutations. Of interest, we occasionally observed off-target effects induced by the 17nt but not the 20nt sgRNAs. These results indicate the importance of balancing on-target gene cleavage potency with off-target effects: when efficacy is a major concern such as genome editing in stem cells, the use of 20nt sgRNAs is preferable.

The clustered, regularly interspaced, short palindromic repeat (CRISPR)-CRISPR-associated 9 (Cas9) system can robustly cleave chromosomal DNA in a targeted manner, producing site-specific DNA double-strand breaks (DSBs). The repair of DSBs induces insertion or deletion mutations (indels) by nonhomologous end-joining (NHEJ), precise gene correction or editing by homology-directed repair (HDR). The most popular CRISPR system uses Cas9 endonuclease from *Streptococcus pyogenes*, which guides through simple base-pair complementarity between the first 20 nucleotides (nt) of an engineered single guide RNA (sgRNA) and a target genomic DNA sequence of interest that lies next to a protospacer adjacent motif (PAM) matching the sequence NGG^1^,^2^,^3^. Thus far, CRISPR-Cas9 has become a simple and highly efficient tool for genome editing in bacteria, yeast and human cells, as well as in whole organisms such as *Dorsophila*, *C. elegans*, zebrafish and mice^4^,^5^,^6^,^7^,^8^,^9^,^10^,^11^,^12^,^13^,^14^. In addition, the genome-wide Cas9/sgRNA lentiviral library has been established as an improved approach for functional genomics studies compared to the shRNA library^15^,^16^,^17^,^18^,^19^,^20^,^21^,^22^,^23^.

Cas9-sgRNA is a powerful genome editing technology; however, unexpected indel mutations are induced at off-target sites that share sequence similarity with the on-target site^24^,^25^,^26^,^27^,^28^,^29^,^30^. Several approaches have been taken to improve the specificity of Cas9-sgRNA, including a paired nicking strategy^24^,^29^,^31^ and dimeric Cas9-based system^32^,^33^. The paired nicking strategy uses two sgRNAs to target adjacent sites on opposite DNA strands, each recruiting a Cas9 variant (Cas9-D10A) that nicks DNA instead of cutting both strands. The truly dimeric Cas9-based system^32^,^33^ requires the dimerization of RNA-guided FokI nucleases (RFNs) for efficient genome editing activity. Both of these approaches require two sgRNAs to make a functional Cas9 nickase pair, and the target sequences must contain two PAM sequences, limiting the choice of target sites. Other methods such as truncation of the 3′ end of sgRNA scaffold^26^ or addition of two guanine nucleotides to the 5′ end of the sgRNA^24^ decrease both the off-target and on-target cleavage efficiency. In addition, the use of recombinant Cas9 protein^34^,^35^,^36^ rather than the Cas9-encoding plasmids can reduce off-target mutations. However, the cost and inconvenience of Cas9 protein limit its wide-spread applications. Recently, a simpler approach has been taken to improve Cas9-sgRNA specificity in 293T cells by truncating sgRNAs from 20nt to 17nt or 18nt^37^. However, it remains unknown whether this conclusion still holds in other types of cells, in particular stem cells, which have potential applications in regenerative medicine. As such, we attempted to evaluate the efficacy and off-targets of 17nt vs. 20nt sgRNAs in induced pluripotent stem cells (iPSCs) and mesenchymal stem cells (MSCs), the two most commonly studied stem cells.

To stringently compare the knockout (KO) efficiency of 17nt vs. 20nt sgRNAs, we established a GFP reporter system, which allows us to accurately measure GFP knockout (GFP-negative cells) by flow cytometry (FACS). We also used a lentiviral vector for Cas9/sgRNA delivery. This vector also expresses a puromycin resistance gene, allowing us to select gene-transduced cells to ~99% by puromycin treatment. Our approach prevents potential artifacts introduced by variable plasmid transfection efficiency in different batches of experiments. With these systems, we confirmed the previous studies in 293T cells showing potent gene knockout with either 20nt or 17nt sgRNAs. However, we found that the 17nt sgRNAs are less efficient than 20nt sgRNAs in gene knockout in iPSCs or MSCs.

## Results

### A GFP-reporter system for studying gene knockout

**Figure 1:**
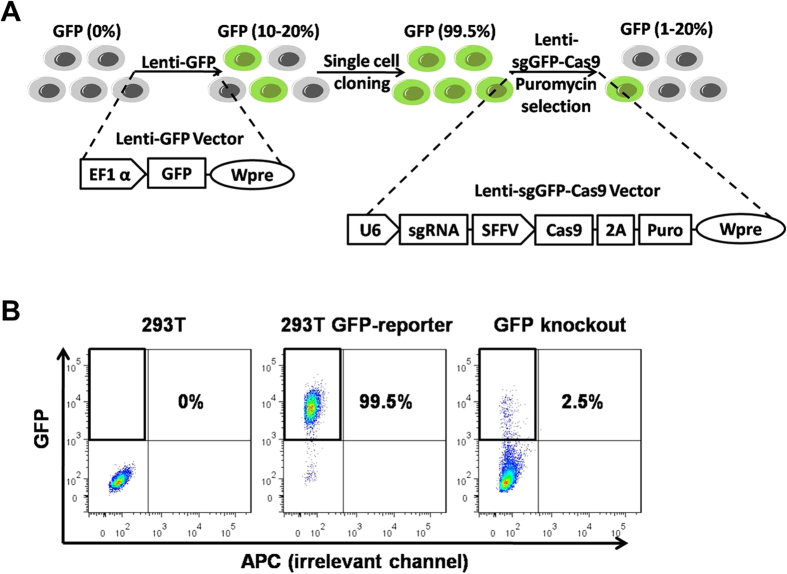
Procedures for establishing GFP reporter cell lines and lentiviral Cas9/sgGFP-mediated gene disruption assay. (**A**) 293T cells, feeder-free human iPSCs or iMSCs (GFP^+^: 0%) were transduced with a lentiviral vector, Lenti-GFP, at a low MOI. After single cell sorting, we picked clones with high-level GFP expression (~99.5%). To knock out GFP, the reporter cell lines were transduced with Lenti-sgRNA-Cas9-puro vectors, in which the U6 promoter drives the expression of sgGFP, and the SFFV promoter drives the expression of both Cas9 and puromycin resistance gene. 2A is a self-cleaving peptide that links 2 genes. Wpre denotes woodchuck hepatitis virus posttranscriptional regulatory element, which stabilizes transcripts and thereby increases gene expression levels. After transduction, repair of Cas9-mediated double-stranded breaks in the GFP reporter gene by error-prone NHEJ-mediated repair leads to frameshift mutations that disrupt the GFP coding sequence, leading to the loss of fluorescence in cells. (**B**) Representative diagrams of FACS analysis of control cells, GFP reporter lines and cells after GFP knockout. Shown are results of 293T cells.

To rigorously investigate the function of truncated 17nt sgRNAs compared to the full-length 20nt sgRNAs, we established a GFP-reporter system, in which GFP was stably expressed in 293T cells by lentiviral transduction at a low multiplicity of infection (MOI) of 0.1–0.2. After single cell sorting, we picked a 293T cell clone that is 99.5% GFP^+^ (Fig. 1) for further expansion and knockout studies.

To knock out GFP, 293T cells were transduced with a sgGFP and Cas9-2A-Puro expressing lentiviral vector at an MOI of approximately one. Starting one to two days after transduction, puromycin was supplemented in the culture medium for ~1 week to select for cells that express relatively high-levels of Cas9 and sgGFP. The Cas9/sgGFP complex then identifies and cleaves the GFP target sequence; repair of double-stranded breaks in the integrated GFP reporter gene by error-prone NHEJ-mediated repair induce frameshift mutations or significant changes in the amino acid sequence, leading to the loss of green fluorescence (Fig. 1A,B).

iPSC and MSC GFP-reporter lines were established similarly. These GFP reporter lines enable us to rapidly and accurately quantify the induction of Cas9-mediated indels by flow cytometry.

### Efficient GFP knockout in 293T-GFP reporter cells mediated by the lentivirally expressed Cas9 and truncated (17nt) or full-length (20nt) sgRNAs

Previous studies used transient transfection system to compare the gene disruption effects of Cas9 with 17nt vs. 20nt sgRNAs^37^. Here we transduced cells with lentiviral vectors followed by puromycin selection, which ensure stable expression of Cas9 and sgRNA in almost 100% of cells. To design optimal sgRNAs, we used the CHOPCHOP program (https://chopchop.rc.fas.harvard.edu/)^38^. We picked sgRNAs with a G (guanine) at 5′ end of sgRNA or tagged a g (guanine), which denotes a mismatched G, to facilitate U6 promoter-mediated transcription.

**Figure 2:**
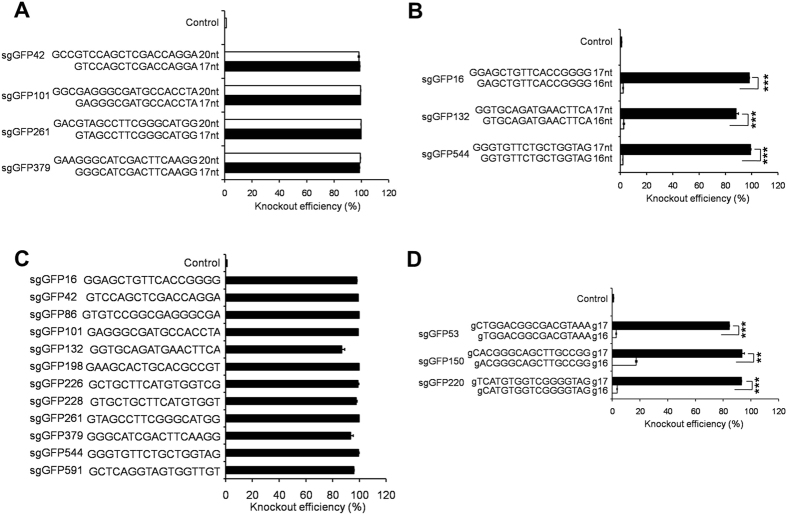
Targeting activities of full-length sgGFPs and truncated sgGFPs in 293T cells. (**A**) Comparison of GFP knockout efficiency of 17nt vs. 20nt sgGFPs in 293T GFP-reporter cells. Four pairs of sgGFPs were designed to target GFP sites 42, 101, 261 and 379. Lengths and sequences of the sgRNAs are shown. Cells transduced with a scrambled sgRNA served as a control. Data shown are mean ± SEM (n = 3). (**B**) Comparison of GFP knockout efficiency of 17nt vs. 16nt sgGFPs in 293T GFP-reporter cells. Three pairs of sgGFPs were designed to target GFP sites16, 132, and 544. Lengths and sequences of the sgRNAs are shown. Data shown are mean ± SEM (n = 3). ***P < 0.001. (**C**) GFP knockout efficiency in 293T GFP-reporter cells with 17nt sgRNAs. Twelve sgGFPs were designed to target different locus of the GFP gene. Data shown are mean ± SEM (n = 3). (**D**) Comparison of GFP knockout efficiency of gN_16_ vs. gN_17_ sgGFPs in 293T GFP-reporter cells. Three pairs of gN_16_ and gN_17_ sgGFPs were designed to target GFP sites 53, 150, and 220. Little g indicates mismatched guanine (G). Sequences of the sgRNAs are shown. Data shown are mean ± SEM (n = 3). **P < 0.01; ***P < 0.001.

To compare the knockout efficiency of truncated and full-length sgRNAs in our system, we designed 4 pairs of 17nt vs. 20nt sgRNAs, with each pair targeting an identical GFP sequence (GFP sites 42, 101, 261 and 379; Fig. 2A). For all of the four pairs, both the 17nt and 20nt sgRNAs showed high-level GFP KO efficiency in 293T cells (>95%) and no differences were observed between the 17nt and 20nt sgRNAs in KO efficiency (Fig. 2A). These data demonstrate that sgRNAs with 17 nucleotides function as efficiently as their matched full-length counterparts in 293T cells.

To investigate whether 17nt is the minimum length for effective sgRNAs, we designed 3 pairs of 17nt vs. 16nt sgRNAs, each targeting an identical GFP sequence (GFP sites 16, 132 and 544; Fig. 2B). sgRNAs with 16nt showed low-level activities (Average: 2%; range: 0.5–5%), whereas their corresponding 17nt sgRNAs generated effective GFP knockout (Average: 93%; range: 84–99%). These results indicate that a minimum length of 17nt is required for a sgRNA to identify and/or cleave its target effectively. To further consolidate this conclusion, we constructed 17nt sgRNAs that target a total of twelve sites on GFP (GFP sites 16, 42, 86, 101, 132, 198, 226, 228, 261, 379, 544 and 591; Fig. 2C). All these 17nt sgRNAs led to high-level GFP disruption (Average: 95%; range: 80–99%). These results demonstrate that truncated 17nt sgRNAs can achieve high-level gene knockout in 293T cells.

Limiting sgRNAs with a matched G at the 5′ end would decrease the availability of optimal sgRNAs by 75%, we thus investigated the effects of tagging a mismatched guanine at the 5′ end. To identify the minimum length of this type of sgRNAs for effective gene disruption, we constructed two versions of sgRNAs: gN_16_ vs. gN_17_, which are 17nt or 18nt in total length, respectively. Three pairs of gN_16_ vs. gN_17_ sgRNAs were designed to target GFP sites 53, 150 and 220 (Fig. 2D). We found that gN_17_ sgGFPs are up to 30-times more efficient than gN_16_ sgGFPs in disrupting GFP (Fig. 2D), suggesting that the minimum length of effective sgRNAs is 18bp when a mismatched g (guanine) is annexed. For all of the 3 gN_17_ sgGFPs, we observed a KO efficiency of 85% ± 3% (Fig. 2D), which is lower than GN_16_ sgGFPs with a matched guanine at the 5′ end (95% ± 3%; *P* < 0.05; Fig. 2C). These data suggest that adding a mismatched g at the 5′ end is an appropriate design for sgRNAs, but the cleavage potency may be slightly lower than sgRNAs with fully matched nucleotides.

### Lower GFP knockout efficiency in iPSCs and iMSCs with 17nt sgRNAs compared to 20nt sgRNAs

In above studies, we observed virtually identical knockout efficiency of 17nt and 20nt sgRNAs in 293T cells. We further asked whether this finding can be reproduced in stem cells. We are particularly interested in iPSCs and MSCs, because iPSCs can be differentiated into all types of cells in the human body for replacement therapy^39^ and MSCs have been used in clinical trials to treat multiple diseases^40^,^41^. In this study, we used iPSCs that were generated from human peripheral blood mononuclear cells^42^ and induced MSCs or iMSCs that were directly reprogrammed from cord blood hematopoietic cells^5^.

**Figure 3:**
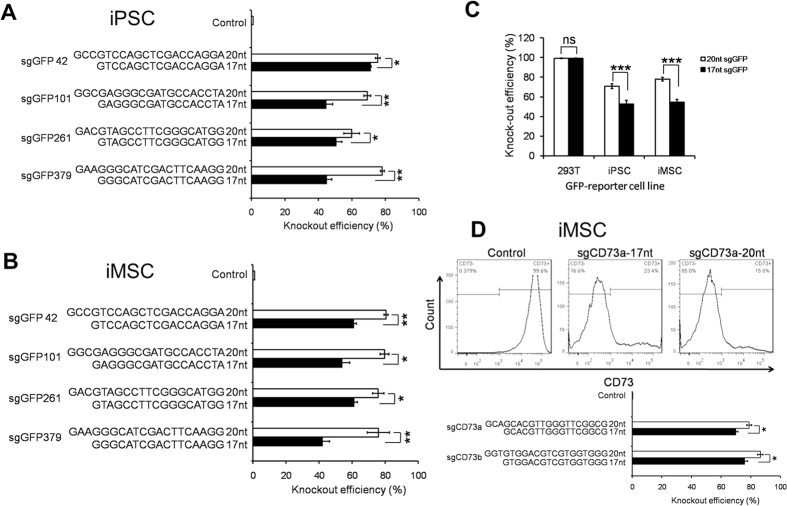
Decreased targeting activities of truncated sgRNAs compared to full-length sgRNAs in iPSCs and iMSCs. Efficiencies of GFP knockout in iPSCs (**A**) and iMSCs (**B**) after transduction of Cas9 and sgRNAs bearing full-length (20nt) or shortened sgRNAs (17nt) that target GFP sites 42, 101, 261 and 379. Lengths and sequences of the sgGFPs are shown. Data shown are mean ± SEM (n = 3). *P < 0.05; **P < 0.01; ***P < 0.001. (**C**) A summary of GFP knockout efficiencies by Cas9 and 17nt vs. 20nt sgGFPs that target 4 sites of the GFP reporter gene (sites 42, 101, 261 and 379) in 293T cells, iPSCs and iMSCs. Data shown are mean ± SEM (n = 4 pairs of sgGFPs). ns, not significant; ***P < 0.001. (**D**) Efficiencies of CD73 knockout in iMSCs after transduction of Cas9 and sgRNAs bearing full-length (20nt) or shortened sgRNAs (17nt) that target the CD73 gene sites a and b. Lengths and sequences of the sgCD73s are shown. Data shown are mean ± SEM (n = 3). *P < 0.05.

To determine the GFP gene disruption efficiency in human iPSCs and iMSCs, we established GFP reporter cell lines using the same approach illustrated in Fig. 1A. We transduced the reporter cells with the same pairs of truncated 17nt sgRNAs and their 20nt counterparts that target GFP sites 42, 101, 261 and 379 (Fig. 3A,B) as showed above (Fig. 2A). To our surprise, in four out of four pairs, we observed a significant decrease in GFP knockout efficiency with 17nt sgRNAs compared to the full-length counterparts. In both iPSCs and MSCs, we observed a reduction of up to 35 percentage points (Fig. 3A,B). Combinatorial analysis of the four pairs of sgRNAs showed that truncated sgRNAs had significantly decreased knockout efficiency in both iPSCs (70% ± 10% vs. 50% ± 5%, *P* < 0.01) and iMSCs (75% ± 8% vs. 52% ± 5%, *P* < 0.01). These data suggest that 17nt sgRNAs may be a good option in 293T cells but 20nt sgRNAs are more potent than truncated sgRNAs in stem cells like iPSCs and iMSCs. Of note, even the 20nt sgGFPs showed significantly decreased gene disruption efficiency in iPSCs (70%) and iMSCs (75%) compared to 293T cells (98%) (Fig. 3C).

To validate the results obtained in GFP-reporter cell lines, we designed two pairs of sgRNA targeting CD73, a surface marker of MSCs. One week after transduction with the Lenti sgCD73/Cas9 vector, the KO efficiency was determined by Anti-CD73 staining and FACS analysis. As expected, for both of the two sgCD73s targeting the coding sequence of the human CD73 gene, 17nt sgRNAs showed significantly lower KO efficiency than the full-length sgRNAs (76% vs. 86% and 70% vs. 79%, *P* < 0.05; Fig. 3D). These results consolidate the conclusion that 17nt sgRNAs are less potent than 20nt sgRNAs in stem cells.

To investigate whether the differences in KO efficiency between the 17nt vs. 20nt sgRNAs and between stem cells and 293T cells are attributable to expression levels of Cas9 and/or sgRNAs, we transduced 293T, iPSCs or iMSCs with lentiviral vectors that express both Cas9-Puro and a 17nt sgRNA or a 20nt sgRNA with a low MOI of 0.3. At 10 days after transduction and puromycin selection, cells were harvested for quantitative real-time RT-PCR analysis. We observed no obvious differences in sgRNA expression in all the cell lines, whereas Cas9 expression levels were ~50% and ~90% lower in iMSCs and iPSCs, respectively, compared to 293T cells (Supplementary Fig. S2). These data suggest that the reduction of KO efficiency in stem cells is most likely due to decreased abundance of Cas9 but not sgRNAs. We also compared expression levels of 17nt and 20nt sgRNAs, and found that similar or even increased sgRNA expression with the truncated version (Supplementary Fig. S2A). Thus, the decreased potency of 17nt sgRNAs in iMSCs and iPSCs cannot be explained by decreased sgRNA expression levels.

### Distinct indel profiles of 20nt and 17nt sgGFPs mediated gene disruption

**Figure 4:**
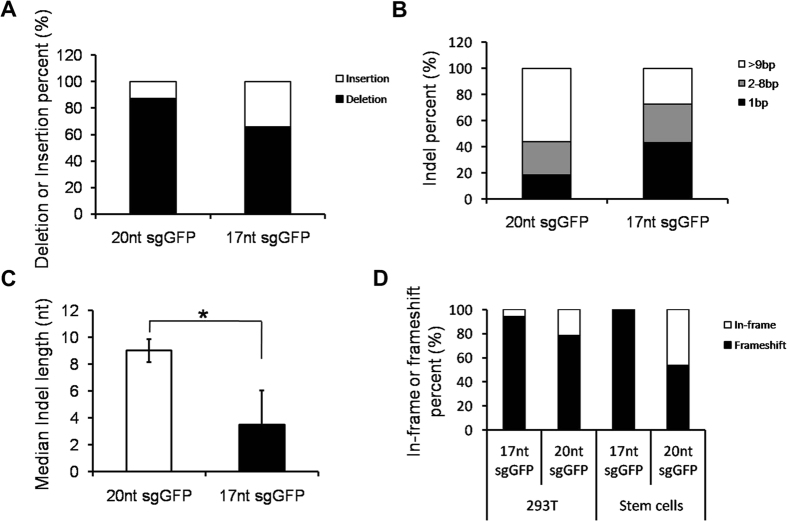
Distinct indel profiles of gene disruptions mediated by 20nt vs. 17nt sgGFP. (**A**) Distribution of deletion and insertion mutations induced by Cas9 and 20nt vs. 17nt sgRNAs targeting four sites of the GFP reporter gene (sites 42, 101, 261 and 379). Detailed information on indels is summarized in Fig. S1. (**B**) Distribution of indel length. The indels were fractionated into 3 groups: 1bp, 2–8 bp, >9 bp. (**C**) Comparison of average indel length induced by Cas9 and 20nt vs. 17nt sgGFPs. (**D**) Comparison of in-frame and frameshift GFP mutations induced by 20nt vs. 17nt sgGFPs. Data shown are mean ± SEM (n = 24). *P < 0.05.

To characterize the indels (nucleotide insertions and deletions) after transduction of Cas9 together with 17nt or 20nt sgGFP, we PCR-amplified the gDNAs flanking the target sequences and conducted Sanger sequencing after cloning of the PCR products (Supplementary Fig. S1A,B). We characterized a total of 99 indels in 293T (34 for 17nt; 42 for 20nt) and stem cells (10 for 17nt; 13 for 20nt). Similarly to previous studies^43^, most indels were deletions (Fig. 4A). However, 17nt sgGFPs led to relatively more small indels (1 bp) and fewer large indels (>9 bp) than their full-length 20nt counterparts (Fig. 4B). In addition, 20nt sgGFPs induced significantly more long indels than 17nt sgGFPs did, resulting in a median indel length increase from ~3.5nt to ~9nt (*P* < 0.05) (Fig. 4C). We also analyzed in-frame vs. frameshift mutations with 20nt vs. 17nt sgRNAs in stem cells and 293T cells. Of interest, we observed more frameshift mutations with 17nt sgGFPs than 20nt sgGFPs (Fig. 4D), which is likely because 17nt sgGFPs induced more single nucleotide mutations (Fig. 4B). These data demonstrate that 20nt sgRNAs induce greater gene disruptions than 17nt sgRNAs, which is consistent with the observation that 20nt sgRNAs are more potent than 17nt sgRNAs in gene knockout.

### Distinct off-target effects of 20nt and 17nt sgGFPs in 293T and stem cells

Finally, we evaluated off-target effects of 20nt vs. 17nt sgGFP in 293T cells, iPSCs and iMSCs. We extracted genomic DNAs from cells transduced with matched full-length (20nt) and truncated (17nt) sgGFPs targeting GFP sites 42, 101, 261 and 379 for analysis. We focused our analysis on 17nt sgGFP induced off-target cleavage. To examine the number of mismatch nucleotides on off-target, we chose four categories of potential off-target sites: (1) 1 mismatch in 17nt: sgGFP42-Off1, sgGFP261-Off1 and sgGFP379-Off3; (2) 2 mismatches in 17nt: sgGFP42-Off2~6, sgGFP101-Off1~7 and sgGFP261-Off3~11; (3) 3 mismatches in 17nt: sgGFP42-Off7 and sgGFP42-Off9; (4) 4 mismatches in 17nt: sgGFP42-Off11~16. We used the standard T7E1 endonuclease cleavage assay to determine DNA disruption at the potential off-target sites (Supplementary Fig. S3A)^44^. The results were also confirmed by Sanger sequencing (Supplementary Fig. S3B), which shows multiple peaks downstream of the predicted Cas9 cleavage site in the histograms. All the results are summarized in Table 1. In 293T cells transduced with 17nt sgGFPs, we detected 2 out of 3 off-targets with 1 mismatch, 6 out of 9 off-targets with 2 mismatches, 0 out of 2 off-targets with 3 mismatches, and 0 out of 5 off-targets with 4 mismatches. These observations suggest that 1–2 mismatches in 17nt sgRNAs can still lead to off-target cleavages, whereas no off-target cleavages are detectable for 17nt sgRNAs with 3–4 mismatches. However, 20nt sgRNAs with even 5 mismatches could also lead to DNA cleavages (sgGFP101-Off7 and sgGFP261-Off7; Table 1).

We then compared off-targets in 293T cells, iPSCs and iMSCs that were transduced with 17nt or 20nt sgGFPs. Among the 29 sites we examined, there were 8 off-targets in 293T cells, whereas only 3 off-targets each for iPSCs and iMSCs in the 17nt sgGFP groups (Table 1). The same trend was also observed for 20nt sgRNAs, with 7 off-targets for 293T cells, 4 off-targets each for iPSCs and iMSCs. These results indicate lower off-target effects in stem cells than in 293T cells, which is in keeping with the lower KO efficiency of sgRNAs in iPSCs and iMSCs. To our surprise, no significant differences were observed in off-target mutations between the truncated (17nt) and the wildtype (20nt) sgRNAs. In one case, 17nt sgRNAs even increased off-target cleavage compared to the 20nt counterpart (sgGFP42-Off1 17nt vs. 20nt; Table 1).

## Discussion

In this study, we accurately measured gene disruption rates of truncated 17nt sgRNAs in comparison with full-length 20nt sgRNAs using a lentiviral-based Cas9/sgRNA vector system and GFP reporter cell lines. With this system, we confirmed that 17nt sgRNAs are indistinguishable from 20nt sgRNAs in knocking out GFP in 293T cells. However, we found that the 17nt sgRNAs are less potent than 20nt sgRNAs in iPSCs and MSCs, possibly in many other types of stem cells and primary cells. We also found that the knockout efficiency of sgRNAs is overall lower in iPSCs and MSCs than in 293T cells, either for truncated or full-length sgRNAs. In association with the decreased potency, we observed significantly lower off-target effects in iPSCs and MSCs compared to 293T cells.

Previous studies using transient transfection showed that 17nt sgRNAs are similar to 20nt sgRNAs in knockout efficiency, but with substantially decreased off-target effects^37^. However, it is unknown whether this conclusion can be extended to cells of significant clinical interest such as stem cells. With this in mind, we used the identical GFP reporter system in 293T cells, iPSCs and iMSCs for stringent comparison. The use of a GFP reporter allows us to accurately measure knockout efficiency by flow cytometry. To prevent the artifacts induced by different transfection efficiency of different batches of experiments, we used lentiviral vectors to express Cas9/Puro/sgRNA and kill off untransduced cells by puromycin treatment. This change also allows us to study iPSCs and iMSCs rigorously, because these cells are difficult to be transfected with plasmids, but can be efficiently transduced with lentiviral vectors.

Using the new system, we found that 17nt and 20nt sgRNAs are virtually identical in their knockout potency in 293T cells, whereas 17nt sgRNAs are significantly less efficient than the 20nt counterparts in iPSCs and iMSCs. The discrepancy between 293T cells and iPSCs/iMSCs can be explained by differential expression levels of Cas9 in different types of cells (Supplementary Fig. S2). 17nt sgRNAs may have decreased binding ability compared to 20nt sgRNAs^37^. However, high levels of the Cas9/ sgRNA ribonucleoproteins in 293T cells might have compensated the lower binding energy of 17nt sgRNAs at the sgRNA/DNA face, thus no difference was observed between 17nt and 20nt sgRNAs in 293T cells. In contrast, in iMSCs and iPSCs whose Cas9 expression levels are ~50% and ~90% lower than those in 293T cells, respectively, lower binding energy of 17nt sgRNAs translated into lower targeting potency compared to 20nt sgRNAs. This can explain the observations that significantly decreased gene disruption rates in iPSCs and iMSCs relative to 293T cells virtually for all the sgRNAs we examined.

Previous reports showed substantially decreased off-target effects of 17nt sgRNAs compared to the full-length 20nt sgRNAs. However, there were seemingly no differences in off-targets between 20nt sgGFPs and 17nt sgGFPs (7–8 out of 29 in 293T cells and 3–4 out of 29 in iPSCs and iMSCs). This apparent discrepancy can be explained by several facts: 1) Our study on off-targets is not comprehensive and we focused our choice on putative off-targets of 17nt sgGFPs; 2) GFP is derived from jellyfish and has less homology with the human genome, which decreases the potential off-target sites. For a typical sgRNA targeting a human gene, the possible off-target sites are often in the hundreds. We showed that 1–2nt mismatches but not 3–4nt mismatches of 17nt sgRNAs can induce off-target cleavage, whereas 20nt sgRNAs with even 5nt mismatches are still effective at some sites. Because there are substantially many more potential off-targets of up to five mismatches for 20nt sgRNAs than those up to two mismatches for 17nt sgRNAs, it is likely that the use of 17nt sgRNAs instead of full-length sgRNAs can substantially decrease off-target effects.

We also investigated the indel profiles. Of interest, we found that cells transduced with 17nt sgGFPs showed substantially more small indels (1 bp) than 20nt sgGFPs (Fig. 4B). The interpretation of these data is that 20nt sgGFPs can still identify and cleave the target DNA with 1nt indel, leading to further gene disruption and thereby decreased the number of small indels and increased the number of large indels^30^.

We observed lower off-targets mutations in iPSCs and iMSCs (3–4 out of 29) than 293T cells (7–8 out of 29), which is in keeping with the whole-genome sequencing analysis that reveals high specificities of CRISPR-Cas9 based genome editing in human iPSCs and ESCs^45^,^46^,^47^,^48^.

Our study also demonstrates the basic design principles for truncated sgRNAs: 1) the shortest length of an effective sgRNA should be 17nt; 2) one mismatched g could be added at the 5′ end of the 17nt matched nucleotides, but the mismatched guanine in the sgRNAs may decrease the targeting efficiency; 3) for sgRNAs with 17nt in length, even one mismatch, such as a mismatched guanine at the 5′ end, markedly decreases on-target efficiency, suggesting the high specificities of 17nt sgRNAs.

In our system, we mainly assessed the gene targeting frequency by loss of GFP based on a lentiviral system. While the use of a lentiviral system is easy as it allows for long term expression, it is possible that some of the GFP loss is due to steric hindrance of GFP transcription. Steric hindrance of gene transcription may have partly contributed to the knockout phenotype. When gene disruption occurs at the genomic DNA level, absolutely no expression will be detected. In contrast, in case of steric hindrance, a low-level appreciable gene expression can be detected, as shown in Fig. 1B. Based on the FACS data, we estimate that steric hindrance of GFP transcription may explain GFP loss in 2–5% of cells. In addition, the use of a heterogeneous GFP^+^ cell population might lead to the relative high background. However, this does not affect the basic conclusion of our study, because the same population of cells was used in all the experimental conditions.

All results above were from a pool of heterogeneous cells transduced with constitutive Cas9/sgRNA expression cassettes randomly integrated into the genome. Therefore, the knockout efficiency and the off-target effects are both expected to be much greater than the transient transfection methods, which most people use. For instance, negligible off-target effects were identified in clonal lines generated after transient transfection with CRISPR/Cas9 plasmids^45^,^46^,^47^.

In conclusion, our results show that in genome editing applications the balance between efficiency and specificity of Cas9-sgRNA mediated cleavage should be considered. We propose that once the targeting efficiency is satisfactory, one may choose truncated sgRNAs, otherwise it is advisable to employ full-length sgRNAs to achieve highest genome editing efficiency.

## Methods

### Lentiviral vectors

The lentiviral vectors used in this study have been described previously^5^,^49^. In these vectors, the EF1 (elongation factor 1 alpha) or SFFV (spleen focus-forming virus long terminal repeat) promoters were used to drive GFP or Cas9 expression, respectively. The details of lentiviral vector packaging and titering have been published elsewhere^50^. In brief, the calcium precipitation method was used for generating lentiviral vectors. After 100-fold concentration by ultracentrifugation, the biological titers of vectors were determined by transducing HT1080 cells.

### sgRNA design

We preferentially picked sgRNAs with a G at the 5′ end, which initiates U6 promoter-mediated transcription and with a G or an A at the 3′ end, which is associated with improved gene targeting efficiency^51^.

### Lenti-U6-sgRNA-SFFV-Cas9-2A-Puro plasmid construction

We used lentiviral plasmid Lenti-U6-sgBbsI-SFFV-Cas9-2A-Puro-Wpre as the sgRNA vector backbone. The vector was digested with BbsI enzyme at 37 °C overnight. For cloning, we synthesized the sgRNA template: TATATATCTTGTGGAAAGGACGAAACACCG N_16–19_ GTTTTAGAGCTAGAAATAGCAAGTTAAAAT. PCR primers are listed as follows: sgRNA-F: TATATATCTTGTGGAAAGGACGAA, sgRNA-R: ATTTTAACTTGCTATTTCTAGCTCTAA. We used the KAPA HiFi polymerase (KAPA BIOSYSTEMS) to amplify the sgRNA product, with the following cycling conditions: 98 °C for 2 min, 1 cycle; 98 °C for 5 sec, 60 °C for 20 sec, 20 cycles. After purifying the PCR products with a QIAquick PCR Purification kit, we assembled 100 ng of the sgRNA backbone and 10 ng of the sgRNA PCR product using Gibson Assembly^®^ Cloning Kit. After transformation, multiple colonies were picked for Sanger sequencing to identify the correct clones. The sequencing primer is U6-F: GGGCAGGAAGAGGGCCTAT.

### Cell culture

293T cells were cultured in DMEM (Dulbecco’s modified Eagle medium; Hyclone) supplemented with 10% fetal bovine serum (FBS; Gibco) and 1% penicillin/streptomycin (ABM). Feeder-free human iPSCs were generated from peripheral blood mononuclear cells, and maintained in E8 medium (Essential 8 medium; Gibco.) in Matrigel-coated (BD) tissue culture plates. Human iMSC were generated from cord blood cells as detailed previously^5^. iMSCs were cultured in Fibronectin (BD)-coated non-tissue culture plates and maintained in α-MEM medium supplemented with 2% FBS, 5% Knockout Serum Replacement (Gibco), 1% ITS, 200 μM ascorbic acid 2-phosphate, and PDGF, EGF and FGF each at 20 ng/ml. 293T and iPSCs were cultured at 37 °C with 5% CO_2_. iMSCs were cultured under hypoxia by placing culture plates in Hypoxia Chambers (Stemcell Technologies, Inc., Vancouver, BC, Canada) that were flushed with mixed air composed of 92% N_2_/3% O_2_/5% CO_2_.

### GFP reporter cell lines

293T cells, feeder-free human iPSCs, and iMSC cells were transduced with lentiviral vector Lenti-EF1-GFP-Wpre at a low MOI of 0.1–0.2. Single cells of GFP-positive cells were sorted into 96-well plates. After 2–3 weeks of culture, cell lines that expressed a stable high-level of GFP were used for knockout studies.

### Mutation rate quantification by GFP-disruption

GFP reporter cell lines, 293T-GFP cells, iPSC-GFP cells and iMSC-GFP cells were transduced with Lenti-U6-sgGFP-SFFV-Cas9-2A-Puro vectors at an MOI of 1 in the presence of 8 μg/ml protamine sulfate. Two days after transduction, cells were treated with 0.5–1 μg/ml puromycin. At 10–12 days following puromycin selection, cells were dissociated with Accutase and analyzed on a BD Arial III flow cytometer. The percentage of GFP negative cells was considered GFP knockout efficiency.

### CD73 disruption assay

iMSCs were transduced with Lenti-U6-sgCD73-SFFV-Cas9-2A-Puro vectors at an MOI of 1 in the presence of 8 μg/ml protamine sulfate. Two days after transduction, cells were treated with 0.5 μg/ml puromycin. At 10–12 days following puromycin selection, cells were dissociated with Accutase and stained with CD73-PE antibody (BioLegend, Inc., San Diego, CA, USA) for 30 min at room temperature. The samples were analyzed on a BD Arial III flow cytometer.

### Sanger sequencing for confirming GFP indel mutations

GFP reporter cells were harvested at 10–12 days after Cas9/sgGFP transduction for DNA extraction using Genomic DNA Extraction Kit (Qiagen). GFP sequence was amplified with KAPA HiFi DNA polymerase by PCR using the following primers, GFP-F: CAGGTGTCGTGAGCGATCGCC, GFP-R: GAACTCCAGCAGGACCATGT. The PCR cycling conditions were 95 °Cfor 4 min followed by 98 °C for 5 sec, 64 °C for 15 sec, 72 °C for 15 sec, 30 cycles. Purified PCR products were then cloned into the pJET1.2/blunt vector using the CloneJET PCR Cloning Kit. The plasmid DNA was transformed into chemically competent Top 10 bacterial cells. Multiple clones were picked for Sanger sequencing. The indels were determined by aligning the sequencing data with the GFP sequence.

### T7EI assay for quantifying frequencies of indel mutation on off-target sites

Potential off-target sites in the human genome were identified using TagScan (http://ccg.vital-it.ch/tagger/tagscan.html)^52^. Cells were harvested at 10–12 days after Cas9/sgGFP transduction. Specific primers were designed with Primer3plus to amplify the sequence flanking the putative off-target sites (Supplementary Table 1). For T7EI mismatch nucleotide cleavage assay, KAPA HiFiDNA polymerase was used to amplify the target sequences using the following conditions: 95 °C for 4 min; 98 °C for 5 sec, 66 °C for 5 sec, 72 °C for 5 sec, 35 cycles. The PCR products were visualized and separated with 1.5% agarose gels and purified with the Thermo PCR product purification kit. Purified PCR products (200 ng) were mixed with 10x NEBuffer2 (New England Biolabs) and nuclease-free water. The DNA was denatured and annealed to form heteroduplexes using the following conditions: 95 °C for 5 min; 95 to 85 °C at −2 °C/sec; 85 °C to 25 °C at −1 °C/sec. One μl of T7 Endonuclease I (New England Biolabs, M0302S) was then added to the annealed PCR products. After incubation for 30 min at 37 °C, the T7E1 reaction was stopped by adding 1.5 μl of 0.25 M EDTA. Cleaved DNA fragments were separated on 2% agarose gels and the percent of nuclease-specific cleavage products (fraction cleaved) was determined by using the ImageJ software. We calculated the percentage of indels using the following formula: % Indel = 100 × (1 − (1 − fraction cleaved)1/2).

### RNA isolation and quantitative real-time RT-PCR

293T cells, iPSCs and iMSCs were transduced with Lenti-U6-sgCD73-SFFV-Cas9-2A-Puro vectors at an MOI of 0.3 in the presence of 8 μg/ml protamine sulfate. Two days after transduction, cells were treated with 0.5–1 μg/ml puromycin for ~1 week. At 10 days after transduction and puromycin selection, cells were harvested by treating with Accutase. Total RNA was extracted using miRCURY RNA Isolation Kit (EXIQON). Reverse transcription was performed using the EasyScript Plus cDNA Synthesis Kit (ABM), following the manufacturer’s instructions. Quantitative real-time RT-PCR (qPCR) was performed as previously described^5^,^50^. Expression of sgRNA and Cas9 was normalized to the expression of GAPDH. The sequences of primers for qPCR are as follows: sgRNA forward, AGCTAGAAATAGCAAGTTAAAATAAGG; reverse, GACTCGGTGCCACTTTTTCA; Cas9 forward, CCGAAGAGGTCGTGAAGAAG; reverse, GCCTTATCCAGTTCGCTCAG; GAPDH forward, GTGGACCTGACCTGCCGTCT; reverse, GGAGGAGTGGGTGTCGCTGT.

### Statistics

Data were analyzed by paired student’s t-test or Wilcoxon test for two groups and ANOVA for more than two groups. All the values were shown as mean ± SEM (standard errors of the mean).

## Additional Information

**How to cite this article**: Zhang, J.-P. *et al*. Different Effects of sgRNA Length on CRISPR-mediated Gene Knockout Efficiency. *Sci. Rep.* **6**, 28566; doi: 10.1038/srep28566 (2016).

## Supplementary Material

Supplementary Information

## Figures and Tables

**Table 1:** Frequencies of indels induced at on-target and off-target sites by 17nt and matched 20nt sgGFPs.

Target ID	Target sequence		293T		iPSC		iMSC	
	20nt	17nt	20nt	17nt	20nt	17nt	20nt	17nt
sgGFP42-On	GCCGTCCAGCTCGACCAGGAtGG	GTCCAGCTCGACCAGGAtGG	98.20%	98.90%	75.50%	71.10%	80.60%	60.87%
sgGFP42-Off1	**TAT**GTCCAGCT**G**GACCAGGAgGG	GTCCAGCT**G**GACCAGGAgGG	26.55%	33.53%	21.40%	26.64%	18.94%	26.79%
sgGFP42-Off2	G**AGC**TCCAGCTCAACCAGGAtGG	**C**TCCAGCTC**A**ACCAGGAtGG	ND	ND	ND	ND	ND	ND
sgGFP42-Off4	G**GGC**TCCAGCTC**A**ACCAGGAtGG	**C**TCCAGCTC**A**ACCAGGAtGG	ND	ND	ND	ND	ND	ND
sgGFP42-Off5	G**TG**GTCCAGCTCG**C**CCAGG**T**cGG	GTCCAGCTCG**C**CCAGG**T**cGG	ND	ND	ND	ND	ND	ND
sgGFP42-Off6	**C**C**T**G**G**CCAGCT**A**GACCAGGAtGG	G**G**CCAGCT**A**GACCAGGAtGG	ND	ND	ND	ND	ND	ND
sgGFP42-Off7	GC**T**G**GA**CAGCTC**T**ACCAGGAtGG	G**G**ACAGCTC**T**ACCAGGAtGG	ND	ND	ND	ND	ND	ND
sgGFP42-Off9	G**G**CG**CA**CAGCTCGACC**T**GGAgGG	G**CA**CAGCTCGACC**T**GGAgGG	ND	ND	ND	ND	ND	ND
sgGFP42-Off10	GCC**TC**CCAGCTC**C**ACCAGG**C**aGG	**TC**CCAGCTC**C**ACCAGG**C**aGG	ND	ND	ND	ND	ND	ND
sgGFP42-Off12	GCC**A**TCCAG**GAG**GACCAGGAtGG	**A**TCCAG**GAG**GACCAGGAtGG	ND	ND	ND	ND	ND	ND
sgGFP42-Off13	GCC**C**TCCA**T**C**C**C**C**ACCAGGAgGG	**C**TCCA**T**C**C**C**C**ACCAGGAgGG	ND	ND	ND	ND	ND	ND
sgGFP42-Off14	GCCGTCCAGCT**CTC**CCAGG**T**gGG	GTCCAGCT**CTC**CCAGG**T**gGG	ND	ND	ND	ND	ND	ND
sgGFP42-Off15	GC**A**GTCCAGCTC**T**A**GG**AGGAaGG	GTCCAGCTC**T**A**GG**AGGAaGG	ND	ND	ND	ND	ND	ND
sgGFP42-Off16	GCCGT**G**CAGCTC**T**A**G**CAGG**G**aGG	GT**G**CAGCTC**T**A**G**CAGG**G**aGG	ND	ND	ND	ND	ND	ND
sgGFP101-On	GGCGAGGGCGATGCCACCTAaGG	GAGGGCGATGCCACCTAaGG	99.50%	99.43%	69.23%	44.40%	79.63%	53.83%
sgGFP101-Off1	G**AGC**AGGG**G**GATGCCACCTAgGG	**C**AGGG**G**GATGCCACCTAgGG	ND	ND	ND	ND	ND	ND
sgGFP101-Off2	G**AT**GAGGG**A**GA**G**GCCACCTAgGG	GAGGG**A**GA**G**GCCACCTAgGG	ND	ND	ND	ND	ND	ND
sgGFP101-Off3	GG**G**GAGGG**A**GAT**T**CCACCTAcGG	GAGGG**A**GAT**T**CCACCTAcGG	21.61%	19.41%	21.72%	21.59%	23.96%	23.77%
sgGFP101-Off5	GG**T**GAGGG**T**GATGCCACC**C**AgGG	GAGGG**T**GATGCCACC**C**AgGG	20.02%	11.49%	ND	ND	ND	ND
sgGFP101-Off6	**AAA**GAGGGC**A**AT**T**CCACCTAcGG	GAGGGC**A**AT**T**CCACCTAcGG	ND	ND	ND	ND	ND	ND
sgGFP101-Off7	**AGG**GAGGGCG**GG**GCCACCTAtGG	GAGGGCG**GG**GCCACCTAtGG	29.91%	29.27%	33.19%	33.15%	33.61%	35%
sgGFP261-On	GACGTAGCCTTCGGGCATGGcGG	GTAGCCTTCGGGCATGGcGG	99.73%	99.80%	50.47%	59.80%	75.77%	61.07%
sgGFP261-Off1	G**CT**G**G**AGCCTTCGGGCATGGcGG	G**G**AGCCTTCGGGCATGGcGG	ND	17.77%	ND	ND	ND	ND
sgGFP261-Off3	G**GA**G**A**AGC**T**TTCGGGCATGGgGG	G**A**AGC**T**TTCGGGCATGGgGG	ND	ND	ND	ND	ND	ND
sgGFP261-Off4	G**GT**GT**G**GCCTT**G**GGGCATGGgGG	GT**G**GCCTT**G**GGGCATGGgGG	ND	ND	ND	ND	ND	ND
sgGFP261-Off5	G**GT**GTAG**G**C**C**TCGGGCATGGcGG	GTAG**G**C**C**TCGGGCATGGcGG	ND	ND	ND	ND	ND	ND
sgGFP261-Off6	**ACA**G**A**AGCCTTC**A**GGCATGGaGG	G**A**AGCCTTC**A**GGCATGGaGG	ND	ND	ND	ND	ND	ND
sgGFP261-Off7	**TTT**GTAG**T**CTTC**A**GGCATGGgGG	GTAG**T**CTTC**A**GGCATGGgGG	11.92%	22.15%	ND	ND	ND	ND
sgGFP261-Off9	**AA**CGTAGCCT**CA**GGGCATGGgGG	GTAGCCT**CA**GGGCATGGgGG	28.64%	17.82%	ND	ND	ND	ND
sgGFP261-Off10	**C**A**G**GTAGCCTT**G**GG**C**CATGGtGG	GTAGCCTT**G**GG**C**CATGGtGG	ND	ND	ND	ND	ND	ND
sgGFP261-Off11	**C**A**T**GTAGCCTTC**A**GGCATG**T**gGG	GTAGCCTTC**A**GGCATG**T**gGG	ND	24.34%	ND	ND	ND	ND
sgGFP379-On	GAAGGGCATCGACTTCAAGGaGG	GGGCATCGACTTCAAGGaGG	99.30%	98.67%	78.17%	44.87%	75.93%	42%
sgGFP379-Off3	**ACG**GGGCATCGA**T**TTCAAGGaGG	GGGCATCGA**T**TTCAAGGaGG	27.44%	ND	23.92	ND	28.71	ND

Nucleotides in bold indicate mismatched target sequences. ND = Not detected.
