# Supplementary material for: Different Effects of sgRNA Length on CRISPR-mediated Gene Knockout Efficiency
Source: Sci Rep. 2016 Jun 24;6:28566. doi: 10.1038/srep28566 (PMC4919781; doi:10.1038/srep28566)
Supplement: Supplementary Information [file srep28566-s1.pdf]

Supplementary Information

Different Effects of sgRNA Length on CRISPR-mediated Gene Knockout Efficiency

Jian-Ping Zhang[1,2*], Xiao-Lan Li[1,2*], Amanda Neises[7], Wanqiu Chen[7], Lin-Ping Hu[1,2], Guang-Zhen Ji[1,2], Jun-Yao Yu[1,2], Jing Xu[1,2], Wei-Ping Yuan[1,2], Tao Cheng[1,2,3,4,5,6], Xiao-Bing Zhang[1,2,3,4,7]

1. State Key Laboratory of Experimental Hematology;
2. Institute of Hematology and Blood Disease Hospital, Chinese Academy of Medical Sciences and Peking Union Medical College, Tianjin, China;
3. Center for Stem Cell Medicine, Chinese Academy of Medical Sciences, Tianjin, China;
4. Department of Stem Cell & Regenerative Medicine, Peking Union Medical College, Tianjin, China;
5. Collaborative Innovation Center for Cancer Medicine, Tianjin, China;
6. Tianjin Key Laboratory of Blood Cell Therapy and Technology, Tianjin, China;
7. Department of Medicine, Loma Linda University, Loma Linda, CA 92350, USA.

*These authors contributed equally to this work.

Correspondences

Xiao-Bing Zhang, PhD. Division of Regenerative Medicine MC1528B, Department of Medicine, Loma Linda University. 11234 Anderson Street, Loma Linda, CA 92350. E-mail: xzhang@llu.edu. Phone: 909-651-5886. Fax: 909-558-0428.

Tao Cheng, MD. State Key Laboratory of Experimental Hematology, Institute of Hematology & Blood Diseases Hospital, Collaborative Innovation Center for Cancer Medicine, Tianjin, China; E-mail: chengtao@ihcams.ac.cn.

Supplementary Figure S1A

293T cells

***sgGFP42*- 17nt sgRNA**

TGAACTTGTGGCCGTTTACGTCGCCGTCCAGCTCGACCA↓GGATGGGCACCACCCCGGTGAACAGCTC wild-type

TGAACTTGTGGCCGTTTACGTCGCCGTCCAGCTC-------ATGGGCACCACCCCGGTGAACAGCTC-7

TGAACTTGTGGCCGTTTACGTCGCCGTCCAGCTCGACC-----GGGCACCACCCCGGTGAACAGCTC-5

TGAACTTGTGGCCGTTTACGTCGCCGTCCAGCTCGAC---GATGGGCACCACCCCGGTGAACAGCTC-3

TGAACTTGTGGCCGTTTACGTCGCCGTCCAGCTCGACC-GGATGGGCACCACCCCGGTGAACAGCTC-1

TGAACTTGTGGCCGTTTACGTCGCCGTCCAGCTCGACC-GGATGGGCACCACCCCGGTGAACAGCTC-1

TGAACTTGTGGCCGTTTACGTCGCCGTCCAGCTCGACCA**c**GGATGGGCACCACCCCGGTGAACAGCT +1

TGAACTTGTGGCCGTTTACGTCGCCGTCCAGCTCGAC**gacgt**CAGGATGGGCACCACCCCGGTGAAC +5

TGAACTTGTGGCCGTTTACGTCGCCGTCCAGCTCGACC**ggcaccgg**AGGATGGGCACCACCCCGGTG +8

***sgGFP42*-20nt sgRNA**

TGAACTTGTGGCCGTTTACGTCGCCGTCCAGCTCGACCA↓GGATGGGCACCACCCCGGTGAACAGCTC wild-type

TGAACTTGTGGCCG-----------------------------GGGCACCACCCCGGTGAACAGCTC -29

TGAACTTGTGGCCGTTTACGTCGCCGTCCAGCTCG---------------ACCCCGGTGAACAGCTC -15

TGAACTTGTGGCCGTTTACGTCGCCGTCCAGCT-------------CACCACCCCGGTGAACAGCTC -13

TGAACTTGTGGCCGTTTACGTCGCCGTCCAGCT-------------CACCACCCCGGTGAACAGCTC -13

TGAACTTGTGGCCGTTTACGTCGCCGTCCAGCTCG------------ACCACCCCGGTGAACAGCTC -12

TGAACTTGTGGCCGTTTACGTCGCCGTCCAGCTC------GATGGGCACCACCCCGGTGAACAGCTC-6

TGAACTTGTGGCCGTTTACGTCGCCGTCCAGCTC------GATGGGCACCACCCCGGTGAACAGCTC-6

TGAACTTGTGGCCGTTTACGTCGCCGTCCAGCTC------GATGGGCACCACCCCGGTGAACAGCTC-6

TGAACTTGTGGCCGTTTACGTCGCCGTCCAGCTCGACC**a**AGGATGGGCACCACCCCGGTGAACAGCT +1

TGAACTTGTGGCCGTTTACGTCGCCGTCCAGCTCGACCAGGA**tacc**TGGGCACCACCCCGGTGAACA +4

TGAACTTGTGGCCGTTTACGTCGCCGTCCAGCTCGAC**gacgt**CAGGATGGGCACCACCCCGGTGAAC +5

TGAACTTGTGGCCGTTTACGTCGCCGTCCAGCTCGACCA**ccccgatgaacagcc**GGATGGGCACCAC +15

TGAACTTGTGGCCGTTTACGTCGCCGTCCAGCTCGAC**gtccatcccgtttacgt**CAGGATGGGCACC +17

***sgGFP101*- 17nt sgRNA**

CAAGTTCAGCGTGTCCGGCGAGGGCGAGGGCGATGCCAC↓CTACGGCAAGCTGACCCTGAAGTTCATC wild-type

<-----------------------------------CACCTACGGCAAGCTGACCCTGAAGTTCATC -83

CAAGTTCAGCGTGTCCG--> -60

<--ACGGCAAGCTGACCCTGAAGTTCATC -55

CAAGTTCAGCGTGTCCGGCGAGGGCGAGGGC---------------AAGCTGACCCTGAAGTTCATC-15

CAAGTTCAGCGTGTCCGGCGAGGGCGAGGGCGATGC--------------TGACCCTGAAGTTCATC -14

CAAGTTCAGCGTGTCCGGCGAGGGCGAGGGCGATGCCAC-------AAGCTGACCCTGAAGTTCATC-7

CAAGTTCAGCGTGTCCGGCGAGGGCGAGGGCGATA-------CGGCAAGCTGACCCTGAAGTTCATC-7

CAAGTTCAGCGTGTCCGGCGAGGGCGAGGGCGATGC----TACGGCAAGCTGACCCTGAAGTTCATC-4

CAAGTTCAGCGTGTCCGGCGAGGGCGAGGGCGATGCCACCT-CGGCAA-CTGACCCTGAAGTTCATC-2

CAAGTTCAGCGTGTCCGGCGAGGGCGAGGGCGATGCCAC-TACGGCAAGCTGACCCTGAAGTTCATC-1

***sgGFP101*-20nt sgRNA**

CAAGTTCAGCGTGTCCGGCGAGGGCGAGGGCGATGCCAC↓CTACGGCAAGCTGACCCTGAAGTTCATC wild-type

CAAGTTCAGCGTGTCCGGCGAGGGCGA-->-189

<------------------------------------CTACGGCAAGCTGACCCTGAAGTTCATC-47

CAAGTTCAGCGTGTCCGGCG------AGGGC--------------------GACCCTGAAGTTCATC-26(-6,-20)

CAAGTTCAGCGTGTCCGG------CGAGGGCGATG-----------------ACCCTGAAGTTCATC -23(-6,-17)

CAAGTTCAGCGTGTCCGGCGAGGGC---------------------AAGCTGACCCTGAAGTTCATC -21

CAAGTTCAGCGTGTCCGGCGAGGGCGAGGGC---------------AAGCTGACCCTGAAGTTCATC-15

CAAGTTCAGCGTGTCCGGCGAGGGCGAGGGCGATG---------GCAAGCTGACCCTGAAGTTCATC-9

CAAGTTCAGCGTGTCCGGCGAGGGCGAGGGCGAT-------ACGGCAAGCTGACCCTGAAGTTCATC -7

CAAGTTCAGCGTGTCCGGCGAGGGCGAGGGCGAT-------ACGGCAAGCTGACCCTGAAGTTCATC-7

CAAGTTCAGCGTGTCCGGCGAGGGCGAGGGCGATGCCAC-TACGGCAAGCTGACCCTGAAGTTCATC-1

***sgGFP261*- 17nt sgRNA**

TGAAGAAGATGGTGCGCTCCTGGACGTAGCCTTCGGGCA↓TGGCGGACTTGAAGAAGTCGTGCTGCTT wild-type

TGAAGAAGATGGTGCGCTCCTGGACGTAGCCTTCG-----GGCGGACTTGAAGAAGTCGTGCTGCTT-5

TGAAGAAGATGGTGCGCTCCTGGACGTAGCCTT----CATGGCGGACTTGAAGAAGTCGTGCTGCTT-4

TGAAGAAGATGGTGCGCTCCTGGACGTAGCCTTCGGGC**a**ATGGCGGACTTGAAGAAGTCGTGCTGCT +1

TGAAGAAGATGGTGCGCTCCTGGACGTAGCCTTCGGGC**a**ATGGCGGACTTGAAGAAGTCGTGCTGCT +1

TGAAGAAGATGGTGCGCTCCTGGACGTAGCCTTCGGGC**a**ATGGCGGACTTGAAGAAGTCGTGCTGCT +1

TGAAGAAGATGGTGCGCTCCTGGACGTAGCCTTCGGGCA**t**TGGCGGACTTGAAGAAGTCGTGCTGCT +1

TGAAGAAGATGGTGCGCTCCTGGACGTAGCCTTCGGGCA**g**TGGCGGACTTGAAGAAGTCGTGCTGCT +1

TGAAGAAGATGGTGCGCTCCTGGACGTAGCCTTCGGGC**tacttga**ATGGCGGACTTGAAGAAGTCGT +7

***sgGFP261*-20nt sgRNA**

TGAAGAAGATGGTGCGCTCCTGGACGTAGCCTTCGGGCA↓TGGCGGACTTGAAGAAGTCGTGCTGCTT wild-type

<--GCGGACTTGAAGAAGTCGTGCTGCTT-106

TGAAGAAGATGGTGCGCTCCTGGACGTAGCCTTCGGGC---------------------------TT-27

TGAAGAAGATGGTGCGCTCCTGGACGTA-------------GCGGACTTGAAGAAGTCGTGCTGCTT -13

TGAAGAAGATGGTGCGCTCCTGGACGTAGCCTTCGGG---------CTTGAAGAAGTCGTGCTGCTT -9

TGAAGAAGATGGTGCGCTCCTGGACGTAGCCTTCG-----GGCGGACTTGAAGAAGTCGTGCTGCTT-5

TGAAGAAGATGGTGCGCTCCTGGACGTAGCCTTCG-----GGCGGACTTGAAGAAGTCGTGCTGCTT-5

TGAAGAAGATGGTGCGCTCCTGGACGTAGCCTTCGGGCA-GGCGGACTTGAAGAAGTCGTGCTGCTT-1

TGAAGAAGATGGTGCGCTCCTGGACGTAGCCTTCGGGCA-GGCGGACTTGAAGAAGTCGTGCTGCTT -1

TGAAGAAGATGGTGCGCTCCTGGACGTAGCCTTCGGGCA**c**TGGCGGACTTGAAGAAGTCGTGCTGCT +1

TGAAGAAGATGGTGCGCTCCTGGACGTAGCCTTCGGGCA**t**TGGCGGACTTGAAGAAGTCGTGCTGCT +1

TGAAGAAGATGGTGCGCTCCTGGACGTAGCCTTCGGGC**a**ATGGCGGACTTGAAGAAGTCGTGCTGCT +1

***sgGFP379*- 17nt sgRNA**

CCCTGGTGAACCGCATCGAGCTGAAGGGCATCGACTTCA↓AGGAGGACGGCAACATCCTGGGGCACAA wild-type

CCCTGGTGAACCGCATCGAGCTGAAGGGCA---------------------ACATCCTGGGGCACAA-21

CCCTGGTGAACCGCATCG---------------------AGGAGGACGGCAACATCCTGGGGCACAA-21

CCCTGGTGAACCGCATCGAGCTGAAGGGCATCGACTTCAAGGA----------ATCCTGGGGCACAA-10

CCCTGGTGAACCGCATCGAGCTGAAGGGCATCGACTTC**a**AAGGAGGACGGCAACATCCTGGGGCACA +1

CCCTGGTGAACCGCATCGAGCTGAAGGGCATCGACTTC**a**AAGGAGGACGGCAACATCCTGGGGCACA +1

CCCTGGTGAACCGCATCGAGCTGAAGGGCATCGACTTC**a**AAGGAGGACGGCAACATCCTGGGGCACA +1

CCCTGGTGAACCGCATCGAGCTGAAGGGCATCGACTTCAA**a**GGAGGACGGCAACATCCTGGGGCACA +1

CCCTGGTGAACCGCATCGAGCTGAAGGGCATCGACTTCA**ggaggggatcgacttc**AGGAGGACGGCA +16

***sgGFP379*-20nt sgRNA**

CCCTGGTGAACCGCATCGAGCTGAAGGGCATCGACTTCA↓AGGAGGACGGCAACATCCTGGGGCACAA wild-type

<-->-291

CCCTGGTGAACCGCATCGAGCTGAAGGGCATCGAC------------------------------->-70

CCCTGGTGAACCGCATCGAGCTG---------------AAGGAGGACG------TCCTGGGGCACAA -21 (-15,-6)

CCCTGGTGAACCGCATCGAGCTGA------------------AGGACGGCAACATCCTGGGGCACAA-18

CCCTGGTGAACCGCATCGAGCTG---------------AAGGAGGACGGCAACATCCTGGGGCACAA-15

CCCTGGTGAACCGCATCGAGCTG---------------AAGGAGGACGGCAACATCCTGGGGCACAA-15

CCCTGGTGAACCGCATCGAGCTG---------------AAGGAGGACGGCAACATCCTGGGGCACAA-15

CCCTGGTGAACCGCATCGAGCTGAAGGGCATCGACTTC----AGGACGGCAACATCCTGGGGCACAA-4

Supplementary Figure S1A. DNA sequences of indel mutations induced by 17nt vs. 20nt sgGFPs that target GFP sites 42, 101, 261 and 379 in 293T GFP reporter cells.

Supplementary Figure S1B

iPSC

***sgGFP101*- 17nt sgRNA**

CAAGTTCAGCGTGTCCGGCGAGGGCGAGGGCGATGCCAC↓CTACGGCAAGCTGACCCTGAAGTTCATC wild-type

CAAGTTCAGCGTGTCCGGCGAGGGCGAGGGCGATGC--------------TGACCCTGAAGTTCATC -14

CAAGTTCAGCGTGTCCGGCGAGGGCGAGGGCGATGC--------------TGACCCTGAAGTTCATC -14

CAAGTTCAGCGTGTCCGGCGAGGGCGAGGGCGATGC----------AAGCTGACCCTGAAGTTCATC -10

CAAGTTCAGCGTGTCCGGCGAGGGCGAGGGCGATGCCAC--ACGGCAAGCTGACCCTGAAGTTCATC -2

CAAGTTCAGCGTGTCCGGCGAGGGCGAGGGCGATGCCAC-TACGGCAAGCTGACCCTGAAGTTCATC -1

CAAGTTCAGCGTGTCCGGCGAGGGCGAGGGCGATGCCACC**c**TACGGCAAGCTGACCCTGAAGTTCAT +1

***sgGFP101*-20nt sgRNA**

CAAGTTCAGCGTGTCCGGCGAGGGCGAGGGCGATGCCAC↓CTACGGCAAGCTGACCCTGAAGTTCATC wild-type

---AAGCTGACCCTGAAGTTCATC -60

CAAGTTCAGCGTGTCCGGCGAGGGCGAGGGCGATGCC------------------CTGAAGTTCATC -18

CAAGTTCAGCGTGTCCGGCGAGGGCGAGGGCGATGC--------------TGACCCTGAAGTTCATC -14

CAAGTTCAGCGTGTCCGGCGAGGGCGAGGGCGATGCCACC------AAGCTGACCCTGAAGTTCATC -6

CAAGTTCAGCGTGTCCGGCGAGGGCGAGGGCGATGCCAC----GGCAAGCTGACCCTGAAGTTCATC -4

CAAGTTCAGCGTGTCCGGCGAGGGCGAGGGCGATGCCAC-TACGGCAAGCTGACCCTGAAGTTCATC -1

CAAGTTCAGCGTGTCCGGCGAGGGCGAGGGCGATGCCAC**gatg**CTACGGCAAGCTGACCCTGAAGTT +4

***sgGFP379*- 17nt sgRNA**

CCCTGGTGAACCGCATCGAGCTGAAGGGCATCGACTTCA↓AGGAGGACGGCAACATCCTGGGGCACAA wild-type

CCCTGGTGAACCGCATCGAGCTGAAGGGCATCGA**gc**T**g**AAGGAGGACGGCAACATCCTGGGGCACAA mutation

CCCTGGTGAACCGCATCGAGCTGAAGGGCATCGACTTC**a**AAGGAGGACGGCAACATCCTGGGGCACAA +1

CCCTGGTGAACCGCATCGAGCTGAAGGGCATCGACTTC**a**AAGGAGGACGGCAACATCCTGGGGCACAA +1

CCCTGGTGAACCGCATCGAGCTGAAGGGCATCGACTTC**a**AAGGAGGACGGCAACATCCTGGGGCACAA +1

CCCTGGTGAACCGCATCGAGCTGAAGGGCATCGACTTC**a**AAGGAGGACGGCAACATCCTGGGGCACAA +1

***sgGFP379*-20nt sgRNA**

CCCTGGTGAACCGCATCGAGCTGAAGGGCATCGACTTCA↓AGGAGGACGGCAACATCCTGGGGCACAA wild-type

CCCTGGTGAACCGCATCGAGCT-------------------GAGGACGGCAACATCCTGGGGCACAA -19

CCCTGGTGAACCGCATCGAGCTGAAGGGCATCGACT------------------TCCTGGGGCACAA -18

CCCTGGTGAACCGCATCGAGCTG---------------AAGGAGGACGGCAACATCCTGGGGCACAA -15

CCCTGGTGAACCGCATCGAGCTGAAGGGCA------TCAAGGAGGACGGCAACATCCTGGGGCACAA -6

CCCTGGTGAACCGCATCGAGCTGAAGGGCATCGACTTC-AGGAGGACGGCAACATCCTGGGGCACAA -1

CCCTGGTGAACCGCATCGAGCTGAAGGGCATCGACTTC**a**AAGGAGGACGGCAACATCCTGGGGCACA +1

CCCTGGTGAACCGCATCGAGCTGAAGGGCATC**gacggcaaggacggcaag**GACTTCAAGGAGGACGG +18

Supplementary Figure S1B. DNA sequences of indel mutations induced by 17nt vs. 20nt sgGFPs that target GFP sites 42, 101, 261 and 379 in iPSCGFP reporter cells.

Supplementary Figure S2

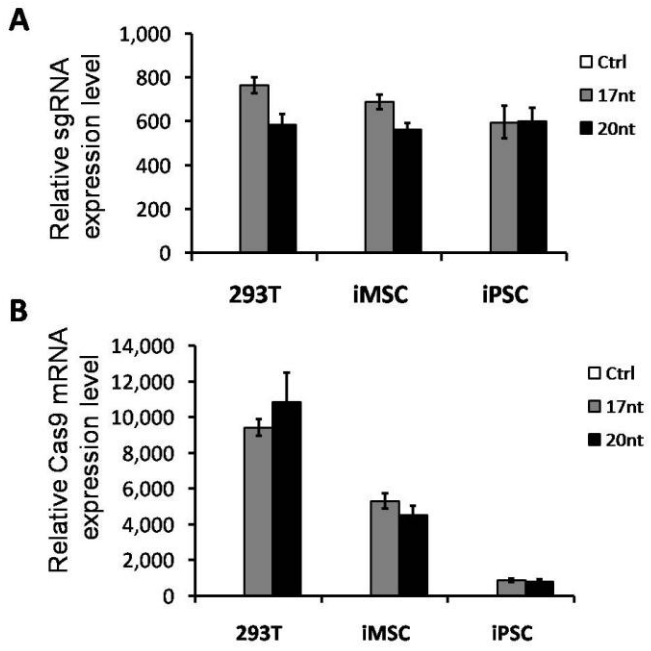

Supplementary Figure S2.Relative expression of 17nt vs. 20nt sgRNA and Cas9 in transduced 293T cells, iMSCs and iPSC. 293T, iPSC and iMSC cells were transduced with Lenti-U6-sgCD73a-SFFV-Cas9-2A-Puro vectors at an MOI of 1 in the presence of 8 µg/ml protamine sulfate. Two days after transduction, cells were treated with 0.5-1 µg/ml puromycin. At 10 days following puromycin selection, cells were harvested by treating with Accutase. Total RNA was extracted using miRCURY RNA Isolation Kit (EXIQON). Reverse transcription was performed using the EasyScript Plus cDNA Synthesis Kit (ABM), following the manufacturer's instructions.

Supplementary Figure S3

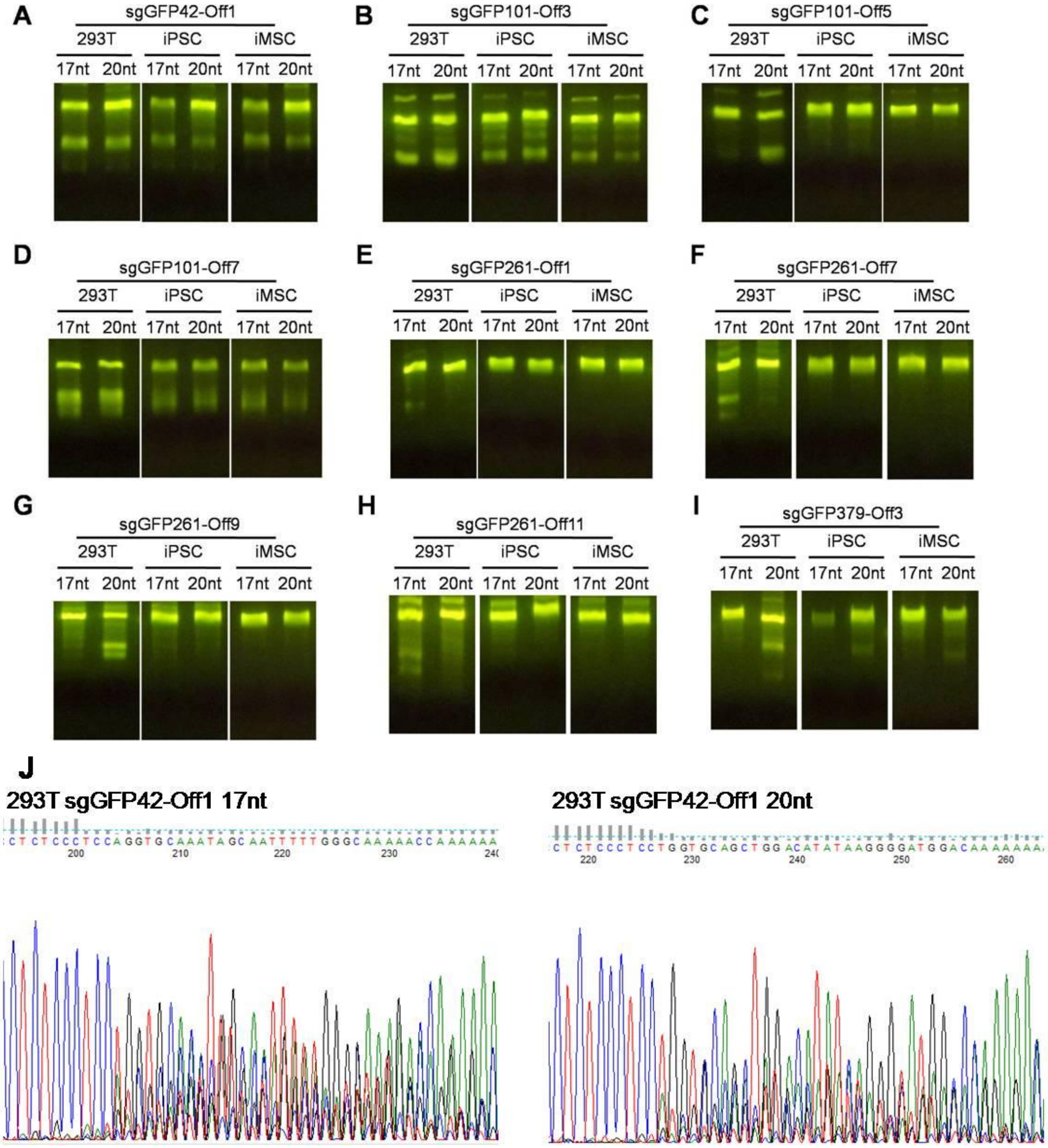

Supplementary Figure S3. Detection of off-targets by T7E1 mismatch nucleotide cleavage assay and DNA sequencing. Gel pictures of off-target amplicons after T7E1 digestion (A-I) and representative histograms of off-target ampliconsequencing (J) are shown.

Table S1. PCR primers used in this study

Name	Forward Primer	Reverse Primer
GFP	CAGGTGTCGTGAGCGATCGCC	GAACTCCAGCAGGACCATGT
sgGFP42-Off1	CCCAGTGACATTGCCCTCTT	CCAGGCTGCTGCTCATCATA
sgGFP42-Off2	ATGGCCAGGCTTCAGTACAC	AGGGTGGGAAGGAAAGGAGA
sgGFP42-Off4	GGGAGTTCGTCCTTTCTGCA	GGTCCCAGGGGAGTTTTTCC
sgGFP42-Off5	AAGCCTTCCAGAACAGCCTC	TGTTCTCAGACGTGGACAGC
sgGFP42-Off6	CCTTTCTCCTGCCCATCTGG	CCACTGTACCACGCTGTCTT
sgGFP42-Off7	CCCAGGCTGCAACATTTGAC	TGACTTGCTAAGAGGGCAGG
sgGFP42-Off9	CTCCAGGACCTGCCAAAGAG	GCCTCCAGGGTTAAGAGCAG
sgGFP42-Off10	ACAGCCCTTGTGTCATCCTC	CTCACTCTGTGCCAGTCCTG
sgGFP42-Off11	CAGTGTGGAACTCGCCTTCT	GAAGCTGAGCCAAGCCCTAA
sgGFP42-Off12	CACCTTGGGGGAAGACTGAC	GGCCCTTCTTCTCTTTGCCT
sgGFP42-Off13	CAGAACCCCTGGACACTGAC	CTCTATGACCACTTGCGGCA
sgGFP42-Off14	CCTGATAGGGAAGCAAGGCC	GCAAATAAGGGGTGGAGGCT
sgGFP42-Off15	GGGTCAGGCCAGGTCTAAAC	TCCTGCCTCACAGCCTATCT
sgGFP42-Off16	TGGAACTGGGGCTCAAATCC	CTGGGGACAATGAGGCTGAG
sgGFP101-Off1	GGGTGGAAGAAAGCACGGTA	CCCCCATTCATCCAGGTCAG
sgGFP101-Off2	AAGGTCTTGGGGCTTTGAGG	AGAACACCCGATTCAGCCTG
sgGFP101-Off3	ACTAAGGTACCAGCCAAACACA	GGGTGAGAGCCAAGGAAACA
sgGFP101-Off4	ACACATGTTCTTGCACGTGC	CTGCCTCACTCTTCTGGCTC
sgGFP101-Off5	TCGGCACTTTGATGAGCACT	GGTTCACCAAGCTCAGGGTT
sgGFP101-Off6	GGATCGCTACAACCCAGGAG	TGCGCTCAAACCATCTTCCT
sgGFP101-Off7	GCCAACCTGGAGAAGTGGAA	ACTCCCATCCTGACTAGTGCT
sgGFP261-Off1	AGACCACAATTCCCGGCATT	CGAACGGGTTCCAGAGTTCT
sgGFP261-Off3	TGTGAGCCCCTCAGAGAGAG	ATGGATCCTCCCCCTCTCAC
sgGFP261-Off4	CAGCTCCATGACCTACTGCC	GTGCCCCTACCTCACATTCC
sgGFP261-Off5	CAAGTCCCCGCTCATCTCAG	AGACCCGGCTCCCTCTATTT
sgGFP261-Off6	GCCTTCTTCGGACCTTCACA	CCTGCAGTAGACTCCCTTGC
sgGFP261-Off7	AGGCTCCCTACTCTGTCCTG	ACCGTTTGTTGCTTGTATGCA
sgGFP261-Off9	CAGCACCCTCCTTCCTTCTG	CCTCAGCCTCCTCCCTTAGT
sgGFP261-Off10	GGCCACGCGTAGGAATTAGA	TCTTCCACAGGGGCAACATC
sgGFP261-Off11	GCACACGGTGTCACATCAAC	CCCAGAAATCCCACCCCATC
sgGFP379-Off3	CGGCCAAGTGGTCAGTTTTC	AGATGGGCCAACTTCTCAGC
sgRNA-qPCR	AGCTAGAAATAGCAAGTTAAAATAAGG	GACTCGGTGCCACTTTTTCA
Cas9-qPCR	CCGAAGAGGTCGTGAAGAAG	GCCTTATCCAGTTCGCTCAG
GAPDH-qPCR	GTGGACCTGACCTGCCGTCT	GGAGGAGTGGGTGTCGCTGT
